# Reflecting on the Implications of Dollar Store Expansions on Food and Nutrition Security in the United States

**DOI:** 10.1016/j.advnut.2024.100337

**Published:** 2024-11-08

**Authors:** Amy R Mobley

**Affiliations:** Department of Health Education and Behavior, University of Florida, Gainesville, FL, United States

Defined by the USDA [1] as the “limited or uncertain availability of nutritionally adequate and safe foods or limited or uncertain ability to acquire acceptable foods in socially acceptable ways safe and affordable food,” food insecurity impacts millions of American households, with higher rates among Black, Hispanic, and rural populations [2]. The definition of nutrition security further emphasizes “equitable access to healthy, safe, and affordable food that promote optimal health and well-being,” by recognizing structural inequities that prevent Americans from eating healthy and being active [3]. Factors associated with food and nutrition insecurity include income, employment, disability, and race and ethnicity [4]. Individuals with food and nutrition insecurity are at increased risk of diet-related diseases such as heart disease and diabetes [5]. For these reasons and more, reducing food and nutrition insecurity is a priority goal of Healthy People 2030, the United States White House, and the FAO of the United Nations [4,6,7]. Specifically, in 2022, President Biden convened the White House Conference on Hunger, Nutrition, and Health to unite nationwide leaders to end hunger and reduce chronic diseases and associated health disparities by 2030 [6].

Further examination of the risk factors and corresponding strategies to improve food and nutrition security are needed. Access to healthy food, such as access to grocery stores, is a social determinant of health and is not equitable across local and regional areas of the United States [8]. Dollar stores have been noted to be one of the fastest growing retailers, especially in rural communities [9] where residents are more likely to experience food and nutrition insecurity [2]. With an increasing presence of dollar stores nationwide and an expansion of food offerings in some stores, a workshop was convened with economists, social scientists, public health researchers, and advocates to further investigate the potential nutrition and public health–related implications on the United States population [10]. The recent article by Feng et al. [10] highlights proceedings of the workshop to identify future research priorities to examine the impacts of increased dollar store presence and food offerings. The insight of workshop organizers and attendees to thoughtfully consider both the positive and potentially negative implications of the expansion of dollar stores should be commended. Key areas of needed research were identified during the workshop and the top categories for research included *1*) health/nutrition; *2*) policy and programs; and *3*) systematic issues. Priority research question topics included *1*) local community impacts; *2*) employees/employment; and *3*) policy and programs [10]. For example, although the added presence of a dollar store to a community with limited food access would seem to be a positive attribute as it relates to health and nutrition, if a dollar store offers limited food options, lower nutrition quality food choices, and/or a lack of culturally preferred food options, improving food and nutrition security will be more challenging. Furthermore, if Supplemental Nutrition Assistance Program or the Special Supplemental Nutrition Program for Women, Infants, and Children benefits are not accepted or result in increased prices for a consumer with limited income, consumer spending power will be reduced. Other potential negative impacts of dollar store entry include a reduction in grocery store sales, increased likelihood of grocery stores closing, and reduced employment all of which are more pronounced in rural communities [11].

Although individuals with lower incomes are more likely to shop at dollar stores compared with conventional supermarkets and club stores, purchases at dollar stores are negatively correlated with healthful food [12]. A prior survey indicated that dollar store consumers have positive views of their presence in a community but desire healthier food options [13]. A recent qualitative study by Reimold et al. [14] aimed to determine motivations for shoppers with low incomes to patronize dollar stores. Over 40% of the participants reported purchasing half of their food at a dollar store. The most common reasons for purchasing food at dollar stores included cost, location convenience, and the food and nonfood items available. Participants reported that dollar stores increased their purchasing power. Although some participants indicated purchasing mainly snack foods from dollar stores, other participants noted a wider variety of purchases including meal and pantry foods and nonfood items. And, similar to prior surveys, participants indicated that they would desire more fresh food options, including fruit, vegetables, and meat.

The highlighted findings and research priorities of the workshop organized by Feng et al. [10] are well aligned with the overall theme of the 2022 White House Conference on Hunger, Nutrition and Health [6]. As a result of the White House Conference on Hunger, a National Strategy containing 5 pillars for government, nonprofit, community, and private agencies to take action to end hunger in the United States was proposed [6]. These 5 pillars include to *1*) improve food access and affordability; *2*) integrate nutrition and health; *3*) empower all consumers to make and have access to healthy choices; *4*) support physical activity for all; and *5*) enhance nutrition and food security research. The increased presence of dollar stores has implications for several of these National Strategy pillars, and as Feng et al. [10] note, there are many unknown impacts of the growing presence of dollar stores in the community as well as food choices provided in these stores that need further research.

Berkowitz et al. [15] recently investigated opportunities to prevent food insecurity rather than address it after it has occurred in the United States population. Their key findings highlight the need for further income supports to prevent food insecurity such as for households with children, disabled, and/or unemployed adults. They conceptualize that food insecurity is not a result of insufficient food production or availability in the United States but to income distribution or lack of purchasing power and therefore, consumption power. Although dollar stores may seem to increase purchase and consumption power, the low cost of purchases can be misleading because the adequacy and nutritional quality of foods available may be lacking.

Importantly, the workshop report organized by Feng et al. [10] contains a roadmap of research priorities to explore the impact of dollar store expansions in communities on nutrition and public health. Recommendations include exploring key impacts on the local community, employee wages, and policies and programs. These collective efforts are a valuable example of the benefits of team science and the careful consideration of how changes in the food environment may impact public health and society in both positive and negative ways. This recommended research has direct implications on understanding the impact of dollar stores or similar retailers on food and nutrition security in the community now and in the future.

## Author contributions

The sole author was responsible for all aspects of this manuscript.

## Funding

The author reported no funding received.

## Conflict of interest

The author reports no conflicts of interest.
